# Pacientes Naïve Infectados por HIV Apresentam Disfunção Concomitante com Diminuição de Anticorpos Naturais contra Autoantígenos Derivados da Apolipoproteína B Definidos

**DOI:** 10.36660/abc.20200062

**Published:** 2021-04-08

**Authors:** Henrique Andrade R. Fonseca, Magnus Gidlund, Viviane Rodrigues Sant’Anna, Esteferson Rodrigues Fernandes, Francisco A. H. Fonseca, Maria Cristina Izar

**Affiliations:** 1 Universidade Federal de São Paulo Escola Paulista de Medicina São PauloSP Brasil Universidade Federal de São Paulo Escola Paulista de Medicina, São Paulo, SP - Brasil; 2 Universidade de Sao Paulo São PauloSP Brasil Universidade de Sao Paulo, São Paulo, SP - Brasil

## Abstract

**Fundamento:**

Fatores de risco definidos para HIV e tradicionais podem estar associados a um aumento de eventos cardiovasculares. Estudos recentes sugerem que a resposta imune humoral à LDL modificada pode estar associada ao processo de aterosclerose.

**Objetivos:**

Avaliar a presença de anti-LDL oxidada e de peptídeos derivados da Apolipoproteína B no sangue, bem como sua associação à função endotelial na infecção por HIV.

**Métodos:**

Este estudo incluiu consecutivamente sujeitos com idade, sexo e dados demográficos correspondentes em dois grupos: (1) indivíduos infectados com HIV e naïve para terapia antiviral e (2) indivíduos não infectados. A aterosclerose subclínica foi avaliada pela espessura íntima-média, utilizando-se a ultrassonografia das artérias carótidas. A função endotelial foi determinada pela dilatação mediada por fluxo (DMF) da artéria braquial por ultrassonografia. Os níveis de autoanticorpos (IgM, IgG) de lipoproteínas de baixa densidade antioxidadas (LDL-ox), fragmentos de peptídeos antiapolipoproteína B (peptídeos ApoB-D e 0033G-Cys), e citocina foram avaliados por meio de ELISA.

**Resultados:**

Os resultados deste estudo não mostraram diferenças na aterosclerose subclínica entre os grupos. Entretanto, os sujeitos infectados com HIV apresentaram uma DMF mais baixa, em comparação com os sujeitos não infectados. Portanto, os sujeitos infectados com HIV apresentaram níveis mais altos de citocinas inflamatórias, títulos de IgG anti-LDL-ox, e IgG anti-ApoB-D. Em contraste, títulos de IgM anti-ApoB-D foram mais baixos em indivíduos infectados com HIV e associados a funções endoteliais diminuídas.

**Conclusões:**

Os resultados deste estudo mostram que a infecção por HIV, em sujeitos naïve, está associada à disfunção endotelial e à diminuição de anticorpos naturais para antígenos Apo-B.

## Introdução

Doenças cardiovasculares são mais prevalentes em indivíduos infectados por HIV, em comparação com indivíduos não infectados.^1^ A disfunção endotelial (DE) é o evento iniciador da formação de placas, associado à inflamação do espaço subendotelial causada pela oxidação da proteína de baixa densidade (LDL).^2^^,^^3^ A detecção de oxidação de LDL pode ser um marcador do processo e/ou do avanço da aterosclerose.^4^

Para superar alguns dos obstáculos relacionados à falta de mais epítopos restritos do que os expressos em um processo de oxidação artificial (cobre, ferro e outros) para gerar a LDL oxidada (LDL-ox), foi determinada a resposta autoimune a peptídeos da Apolipoproteína B (ApoB) - derivada de uma partícula de LDL. Estudos anteriores demonstraram que anticorpos contra um peptídeo específico (ApoB-D) podem ser considerados um marcador de ativação inflamatória.^5^^–^^7^ Entretanto, não foi demonstrado que infecções crônicas podem modular os autoanticorpos (AAC) em autoantígenos, especialmente na condição de deficiência do sistema imune.

## Materiais e métodos

### Sujeitos

Este trabalho conduziu um estudo piloto caso-controle transversal que incluiu prospectivamente 40 sujeitos infectados por HIV, naïve para terapia antirretroviral altamente ativa (HAART, do inglês *highly active antiretroviral therapy*), de ambos os sexos. Cinquenta e três sujeitos não infectados com HIV (controle) foram recrutados nas mesmas comunidades, utilizando-se os mesmos anúncios e fatores de risco cardiovasculares. Depois de fazer a coleta de sangue e avaliações clínicas, os pacientes infectados com HIV que iniciaram a HAART aderiram à medicação prescrita.

### Lipídios e análise bioquímica

O colesterol total sérico, o colesterol de lipoproteína de alta densidade (HDL-C), e os triglicérides foram determinados enzimaticamente (Opera Bayer, Leverkusen, Alemanha), com o colesterol de lipoproteína de baixa densidade (LDL-C) estimado pela equação *Friedewald* quando os triglicérides eram <400 mg/dl.^8^ A glicose foi avaliada pelo método enzimático.

### Função endotelial e espessura íntima-média da carótida

Testes de ultrassom foram realizados para avaliar a aterosclerose subclínica por meio da espessura íntima-média da carótida (EIMC)^9^ e avaliação de vasorreatividade da dilatação mediada por fluxo (DMF) dependente do endotélio da artéria braquial.^10^

Brevemente, os pacientes tiveram que fazer jejum e não consumir nitratos e álcool, e não fazer uso de medicamentos vasoativos 24 horas antes dos exames. Depois de um repouso de 15-20 minutos, a artéria braquial da fossa antecubital direita foi visualizada, utilizando-se um transdutor linear com frequência de até 11 MHz, com monitoramento simultâneo por eletrocardiograma (ECG). As imagens foram obtidas pelo sistema de ultrassom HP SONOS 5500 (Hewlett Packard, Palo Alto, EUA). Quanto foi obtida a imagem ideal da artéria, o diâmetro do vaso de linha de base foi medido. Foi induzida a hiperemia reativa inflando-se o manguito até 200 mmHg, ou pelo menos 50 mmHg acima da PAS, no antebraço distal por 5 minutos e, em seguida, esvaziando-se o manguito. Imagens diastólicas finais foram obtidas no momento do surgimento do complexo QRS no ECG. Essas imagens foram capturadas na linha de base e um minuto após o esvaziamento da braçadeira. A mudança de porcentagem do diâmetro da linha de base até o valor detectado durante a hiperemia reativa foi calculada para determinar a DMF. As medidas de DMF e a EIMC foram avaliadas por um ultrassonografista experiente de maneira cega. As variabilidades de resultados do próprio sonógrafo e entre sonógrafos foram menores que 1% e 2%, respectivamente.

### Citocinas e células T CD4

As concentrações de citocina foram testadas utilizando-se kits ELISA disponíveis comercialmente. A carga viral plasmática e as contagens de células T CD4 foram determinadas para os sujeitos infectados com HIV. O nadir de células T CD4+ foi definido como o valor mais baixo registrado e confirmado em laboratório.

### Isolamento e síntese de autoantígeno

A partícula de LDL foi obtida do plasma total após a centrifugação (1.000 g; 4 °C; 15 min) e suplementada com benzamidina (2 mM), gentamicina (0,5%), cloranfenicol (0,25%), fluoreto de fenilmetilsulfonil (PMSF) (0,5 mM), e aprotinina (0,1 unidade/mL). Partículas de lipoproteína de baixa densidade (1,006<d<1,063 mg/mL) foram isoladas por ultracentrifugação sequencial (100.000 g; 4 °C), utilizando-se um rotor (70 Ti, ângulo fixo; Beckman Coulter, EUA) e uma ultracentrífuga (Hitachi, Japão). A partícula de LDL foi oxidada por cobre e utilizada como autoantígeno para avaliar os títulos de anticorpos.^11^ Os peptídeos de Apolipoproteína B (peptídeos ApoB) usados neste estudo foram compostos de dois fragmentos sintéticos: ApoB-D (ApoB-D, que é um fragmento de peptídeo ApoB com uma sequência de 22 aminoácidos derivados do domínio 3 da sequência da Apolipoproteína B na terceira porção conservada para digestões de tripsina),^12^ e o peptídeo-0033G-Cys (peptídeo-0033G-Cys, que é um fragmento de peptídeo com uma sequência de 21 aminoácidos derivados do domínio 3 da sequência da Apolipoproteína B na primeira porção conservada para digestão de tripsina).^12^

### Determinações de autoanticorpos

A quantificação da LDL-ox e dos autoanticorpos (AAC) derivados do peptídeo ApoB foi avaliada no plasma total por ELISA, conforme descrito anteriormente.^13^^,^^14^ Microplacas de 96 poços (Microplates 8096, Costar-EUA) foram cobertas com 10µg/mL do peptídeo ApoB-D ou 0033G-Cys em tampão carbonato/bicarbonato (0,1 mol/l; pH 9,6), que foi deixado em sensibilização durante a noite a 4ºC. Depois de três ciclos de lavagem com solução salina tamponada com fosfato (PBS, pH 7,4) mais Polissorbato-20 (0,05%), a placa foi bloqueada com gelatina (3%; temperatura ambiente; 24 h). Amostras de plasma dos pacientes (50 μl/poço, 1:400 em tampão fosfatado, PBS, pH 7,4) foram acrescentadas às placas por 2 horas em temperatura ambiente. Em seguida, três ciclos adicionais de lavagem foram realizados, e foram acrescentados anticorpos conjugados com peroxidase horseradish de IgG secundário (anticorpo de cabra anti-IgG de humano, 0,1 µg/ml, KPL, Kirkegaard & Perry Laboratories, Gaithersburg, Maryland, EUA) ou IgM (anticorpo de cabra anti-IgM de humano purificado, 10 µg/ml, KPL, Kirkegaard & Perry Laboratories, Gaithersburg, Maryland, EUA), para avaliar os títulos de AAC de peptídeos anti-ApoB-D ou anti-0033G-Cys. Depois da incubação (1 hora), as placas foram lavadas (três ciclos), e foram acrescentados 3,3,5,5-tetrametilbenzidina (6,5% in dimetilsulfóxido; Sigma, St Louis, MO) e H_2_O_2_ (Sigma) diluídos em tampão fosfato-citrato (0,1mol/l; 250μl; pH 5,5) em temperatura ambiente, como enzima-substratos. A reação foi interrompida acrescentando-se H_2_SO_4_ (2mol/l). A densidade ótica (DO) das amostras foi medida em 450 nm. Títulos de autoanticorpos (AAC) foram expressos como índice de reatividade (IR), calculado como $\text{IR}=({\text{DO}}_{\text{amostra}}–{\text{DO}}_{\text{amostra em branco}})/({\text{DO}}_{\text{IgG ou IgM}}–{\text{DO}}_{\text{IgG ou IgM em branco}})$ em que os anticorpos IgG ou IgM foram usados como controles. O coeficiente de variação intraensaios foi de 5,4% e o intraensaios foi de 2,0%.

Foram realizados títulos de AAC Anti-LDL-ox similares para o ensaio com peptídeo de Apolipoproteína B, utilizando-se, contudo, microplacas de noventa e seis poços cobertos com 7,5 μg/ml de LDL-ox.^13^ Os anticorpos totais foram determinados em plasma total pelo método ELISA.

As amostras foram executadas triplicadas e a variação entre os triplos não ultrapassou 5% da média.

### Ética

O estudo foi aprovado pelo comitê de ética institucional da Universidade Federal de São Paulo e da Universidade de São Paulo (FO. 99/2009), e todos os participantes assinaram termos de consentimento informado antes do início do protocolo.

### Análise estatística

As análises estatísticas foram realizadas utilizando-se o pacote de software SPSS 17.0 (Statistical Package for Social Science, SPSS Inc., Chicago, IL, EUA). As variáveis categóricas foram comparadas por meio do teste qui-quadrado de Pearson. A distribuição de normalidade foi avaliada pelo teste de Kolmogorov-Smirnov. As análises entre grupos foram testadas por teste *t* ou teste Mann-Whitney. A interação entre a função endotelial e outras variáveis foram testadas com os testes de Pearson ou Spearman. Variáveis cuja interação foi identificada como significativa foram testadas com análise de regressão linear múltipla stepwise, com a função endotelial como variável dependente. Foi usado um nível de significância de 5% para todos os testes.

## Resultados

Os parâmetros clínicos e demográficos são apresentados na Tabela 1. Não houve diferença de EIMC entre sujeitos infectados com HIV e não infectados. A função endotelial foi diminuída em sujeitos infectados com HIV (p=0,040). (Tabela 1).

**Tabela 1:** Características dos sujeitos infectados com HIV naïve e não infectados

Variáveis		Geral (93)	HIV- (Controle) (53)	HIV+ (Naïve) (40)	p-valores
**Parâmetros clínicos**					
	Sexo (masculino/feminino)	63/30	32/21	31/9	0,110
	Idade (anos)	32 (1,0)	32 (1,7)	32 (1,3)	0,746
	Circunferência abdominal (cm)	88 (83-97)	89,5 (76,5-100)	97 (83-96)	0,668
	Índice de Massa Corporal (kg/m²)	24,8 (23-28)	25,5 (21,5-28,5)	25,2 (23,5-28)	0,586
	Fumantes (%)	11	6	5	0,951
	Pressão sanguínea sistólica (mmHg)	120 (110-120)	120 (110-120)	120 (110-120)	0,631
	Pressão sanguínea diastólica (mmHg)	80 (70-80)	80 (70-80)	80 (70-80)	0,441
**Análise bioquímica**					
	Colesterol total (mg/dL)z	165 (139-185)	166 (144-191)	150 (124-176)	0,028
	LDL-c (mg/dL)	98 (69-115)	103 (77-119)	91 (66-113)	0,095
	HDL-c (mg/dL)	46 (37-65)	47 (40-57)	36 (30-46)	0,008
	Triglicérides (mg/dL)	91 (52-122)	88 (67-131)	113 (73-131)	0,285
	Glicose (mg/dL)	90 (86-94)	90 (86-94)	92 (86-96)	0,425
**Parâmetros de infecção por HIV**					
	Tempo de infecção (anos)	–	–	3 (1-6)	N.A
	Contagem de células CD4 (células/µL)	–	–	447 (366-590)	N.A
	Nadir CD4 (células/µL)	–	–	402 (356-537)	N.A
	Carga viral de HIV (cópias de RNA/µL)	–	–	2623 (485-26225)	N.A
	Coinfecção por HBV	0	0	4	N.A
	Coinfecção por HCV	0	0	3	N.A
**Terapia em uso**					
	Anti-hipertensivos (indivíduos, N)	4	3	1	N.A
	Estatinas (indivíduos, N)	0	0	0	N.A
	Drogas neurológicas (indivíduos, N)	3	2	1	N.A
**Marcadores inflamatórios**					
	hs-CRP (mg/L)	1,20 (0,30-1,92)	0,51 (0,20-1,87)	1,48 (0,82-3,30)	0,017
	IFN-γ (pg/dL)	2,84 (0,90-6,85)	1,43 (0,87-4,10)	3,89 (1,30-8,85)	0,021
	TNF-α (pg/dL)	6,66 (5,58-7,31)	6,02 (5,51-6,94)	6,90 (6,54-7,63)	0,020
	IL-6 (pg/dL)	1,54 (1,37-1,80)	1,54 (1,36-1,63)	1,50 (1,37-1,95)	0,028
	IL-8 (pg/dL)	3,13 (2,50-4,60)	2,80 (2,20-4,40)	3,65 (2,70-5,50)	0,050
	IL-10 (pg/dL)	1,75 (0,39-1,97)	1,79 (0,80-1,98)	0,87 (0,36-1,94)	0,088
**Anticorpos totais**					
Total sérico de IgG (IR)		1,33 (1,19-1,38)	1,34 (1,20-1,38)	1,33 (1,18-1,37)	0,877
Total sérico de IgM (IR)		0,69 (0,55-0,84)	0,67 (0,49-0,82)	0,3(0.58-0.86)	0,310
**Aterosclerose subclínica**					
	Espessura íntima-média (mm)	0,67 (0,57-0,68)	0,67 (0,56-0,68)	0,67 (0,57-0,68)	0,971
**Função endotelial**					
	Dilatação mediada por fluxo (%)	11,6 (1,4)	13,7 (2,4)	9,3 (1,2)	0,040

HBV: vírus da hepatite B; HCV: vírus da hepatite C; N.A: não se aplica; IR: índice de reatividade.

Como esperado, sujeitos infectados com HIV tinham níveis de marcador inflamatório significativamente mais altos do que indivíduos não infectados. Entretanto a citocina anti-inflamatória IL-10 não variou entre os grupos (Tabela 1).

**Figura 1:**
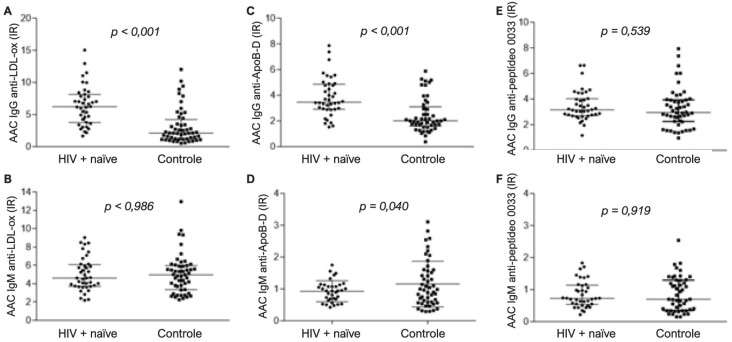
Resposta humoral a LDL oxidada, peptídeo ApoB-D e peptídeo 0033 em pacientes infectados com HIV e controles não infectados. (A) Autoanticorpos (AAC) IgG anti-LDL-ox; (B) AAC IgM anti-LDL-ox; (C) AAC IgG peptídeo anti-ApoB-D; (D) AAC IgM anti-ApoB-D. (E) AAC IgG peptídeos anti-0033; (F) IgM peptídeos anti-0033. Diferenças significativas entre os grupos foram calculadas pelo teste de Mann-Whitney.

Os títulos de AAC IgG e IgM totais séricos não variaram entre sujeitos infectados e não infectados com HIV (Tabela 1). A Figura 1 demonstrou que os títulos de AAC de IgG anti-LDL-ox eram mais altos em sujeitos infectados por HIV (p<0.001). Entretanto, os títulos de AAC de IgM anti-LDL-ox não variaram entre sujeitos infectados e não infectados com HIV. Os sujeitos infectados com HIV tiveram títulos mais altos de AAC de IgG anti-ApoB-D (p<0,001) e títulos mais baixos de IgM anti-ApoB-D em comparação com sujeitos não infectados (p=0,040). Nenhuma diferença foi encontrada entre os grupos em relação aos AAC de antipeptídeo-0033G-Cys.

O presente estudo demonstrou que, em sujeitos infectados com HIV, a função endotelial foi associada aos títulos IgM anti-ApoB-D AAC [β=10,75; p=0,015] (Tabela 2). O modelo de regressão stepwise, incluindo fatores de risco cardiovascular tradicionais, marcadores relacionados ao HIV, e respostas imunes, mostrou que AAC IgM anti-ApoB-D estavam associados à função endotelial [β=7,28; p=0,002]. Não foram observadas associações entre IgG anti-ApoB-D e a função endotelial. Em relação à aterosclerose subclínica, as medidas de EIMC não foram associadas à resposta humoral para ambos os peptídeos.

**Tabela 2:** Análise univariada ajustada de possíveis fatores de risco associados à função endotelial em sujeitos infectados com HIV

Variáveis		
	β	p-valores
Idade (anos)	-0,187	0,350
Circunferência abdominal	-0,049	0,708
IgM peptídeo anti-ApoB-D	10,754	0,015
IgG peptídeo anti-ApoB-D	0,597	0,351
Nadir CD4	0,007	0,135
CD4 atual	-0,010	0,126
Carga viral registrada	0,413	0,786
Tempo de infecção	-0,215	0,718

O coeficiente β representa as alterações na porcentagem de dilatação mediada por fluxo nas variáveis preditoras. Foram feitos ajustes para hipertensão, tabagismo atual, e dislipidemia. IC: Intervalo de confiança.

## Discussão

O presente estudo mostrou que, em sujeitos infectados por HIV, naïve de terapia antirretroviral, uma função endotelial reduzida acompanha uma modulação distinta em AAC contra fragmentos de peptídeos ApoB, em comparação com sujeitos não infectados, independentemente dos títulos de AAC com totais séricos.

Dados relacionados a imunidade humoral de peptídeos ApoB sugerem que sua presença está associada ao avanço da doença aterosclerótica, como parte de uma resposta autoimune.^5^^,^^14^ Entretanto, esses AAC podem participar de eliminação de produtos pró-aterogênicos gerados a partir da oxidação de partículas LDL, e da modificação dos ApoB, desempenhando uma função dupla no processo de aterogênese.^15^^,^^16^

Este estudo também mostrou que AAC de IgM contra ApoB-D estavam associados à ED, corroborando com estudos anteriores.^6^^,^^17^ Nossos achados sugerem que há uma eliminação de autoantígenos ApoB por anticorpos naturais, sugerindo que eles possam estar envolvidos em reparos vasculares após um processo de lesão,^18^ entretanto, os efeitos da infecção por HIV da DMF podem ser atribuídos a um estágio distinto da doença e às diferentes terapias medicamentosas adotadas.^19^

Estudos de coorte e meta-análise demonstraram que a EIMC é mais alta em pacientes infectados com HIV, em comparação com não infectados.^20^ Acreditamos que o momento da infecção em nosso estudo não foi suficiente para promover modificações ateroscleróticas na carótida, detectadas por exame de ultrassom.

Os resultados do presente estudo sugerem que autoanticorpos para peptídeos definidos-ApoB podem ser um marcador de disfunção endotelial, ou mesmo de uma resposta inflamatória elevada, porém não de aterosclerose da carótida em pacientes infectados com HIV. Estudos clínicos coorte em pacientes submetidos a HAART merecem investigação adicional para confirmar esses resultados preliminares.

O desenho transversal e a falta de um grupo que estivesse recebendo tratamento antirretroviral para permitir comparações dos efeitos das drogas HAART na função endotelial e na aterosclerose subclínica são limitações. Nenhuma diferença significativa foi encontrada entre os sexos, o que pode ser justificado pelo pequeno número de sujeitos incluído neste estudo. Estudos adicionais, incluindo um número maior de pacientes, são necessários para confirmar nossos achados em relação a sexo, infecção e função endotelial. Para fins de ajuste, os efeitos de fatores de risco cardiovascular diferentes e marcadores de infecção foram avaliados como uma possível explicação para a resposta imune natural observada, associada à função vascular.

## Conclusão

Os achados deste estudo sugerem que a imunidade natural a antígenos ApoB está associada à DE. Mais estudos prospectivos são necessários para a avaliação de parâmetros imunológicos de HIV em resposta autoimune e seus efeitos na função vascular.
