# Supplementary material for: Pacientes Naïve Infectados por HIV Apresentam Disfunção Concomitante com Diminuição de Anticorpos Naturais contra Autoantígenos Derivados da Apolipoproteína B Definidos
Source: Arq Bras Cardiol. 2021 Apr 8;116(4):844–9. [Article in Portuguese] doi: 10.36660/abc.20200062 (PMC8121411; doi:10.36660/abc.20200062)
Supplement: Translation

# Translation: HIV-Infected Naïve Patients Exhibit Endothelial Dysfunction Concomitant with Decreased Natural Antibodies Against Defined Apolipoprotein B Autoantigens

**Authors:** Henrique Andrade R. Fonseca, Magnus Gidlund, Viviane Rodrigues Sant’Anna, Esteferson Rodrigues Fernandes, Francisco A. H. Fonseca, Maria Cristina Izar

**Affiliations:** 1 São PauloSP Brazil Universidade Federal de São Paulo Escola Paulista de Medicina, São Paulo, SP - Brazil; 2 São PauloSP Brazil Universidade de Sao Paulo, São Paulo, SP - Brazil

**Keywords:** HIV Infection, Atherosclerosis, Endothelium Vascular, Apolipoproteunes B, Carotid Arteries/ultrasonography

## Abstract

**Backgorund:**

Traditional and HIV-defined risk factors may be associated with an increase in cardiovascular events. Recent studies have suggested that the humoral immune response to modified LDL may be associated with the process of atherosclerosis.

**Objectives:**

To evaluate the presence of anti-oxLDL and apolipoprotein B-derived peptides in the blood, and their association with the endothelial function in HIV-infection.

**Methods:**

This study consecutively included subjects matched for age, gender, and demographic data in two groups: (1) HIV-infected and naïve for antiviral therapy and (2) uninfected individuals. Subclinical atherosclerosis was assessed by intimal-media thickness, using ultrasonography of the carotid arteries. Endothelial function was determined by flow-mediated dilatation (FMD) of the brachial artery by ultrasonography. Autoantibodies (IgM, IgG) anti-oxidized low-density lipoprotein (oxLDL), anti-apolipoprotein B-peptide fragments (ApoB-D and 0033G-Cys peptides), and cytokine levels were evaluated by ELISA.

**Results:**

This study's results showed no difference in subclinical atherosclerosis between groups; however, HIV-infected subjects showed a lower FMD, when compared to non-infected subjects. Therefore, HIV-infected subjects showed higher levels of inflammatory cytokines, titers of IgG anti-oxLDL, and IgG anti-ApoB-D. In contrast, titers of IgM anti-ApoB-D were lower in HIV-infected individuals and associated with reduced endothelial functions.

**Conclusions:**

This study's results show that HIV infection, in naïve subjects, is associated with endothelial dysfunction and a decline of natural antibodies to apo-B antigens.

Cardiovascular disease is more prevalent in HIV-infected, as compared to non-infected, individuals.^1^ Endothelial dysfunction (ED) is the initiating event in plaque formation, associated with sub-endothelial space inflammation caused by low density lipoprotein (LDL) oxidation.^2^^,^^3^ Detection of LDL oxidation can be a marker of atherosclerosis processes and/or progression.^4^

To overcome some of the obstacles related to the lack of more restricted epitopes than those expressed in an artificial oxidation process (copper, iron, and others) to generate oxidized LDL (oxLDL), the autoimmune response to apolipoprotein B (apoB) peptides-derived from an LDL particle was determined. Previous studies showed that antibodies against a specific peptide (ApoB-D) can be considered a marker of inflammatory activation.^5^^–^^7^ However, it has not been shown that chronic infection can modulate the autoantibodies (Abs) into auto-antigens, especially in the immune system deficiency condition.

## Materials and methods

### Subjects

This study conducted a cross-sectional, case-control, pilot study including prospectively 40 HIV-infected subjects, naïve for highly active antiretroviral therapy (HAART) for both genders. Fifty-three HIV non-infected subjects (control) were recruited from the same communities, using the same advertisements and cardiovascular risk factors. After blood collections and clinical evaluations, HIV-infected patients who began HAART therapy adhered to the prescribed medications.

### Lipids and biochemical analysis

Serum total cholesterol, high-density lipoprotein cholesterol (HDL-C), and triglycerides were determined enzymatically (Opera Bayer, Leverkusen, Germany) with low-density lipoprotein cholesterol (LDL-C), estimated by the *Friedewald* equation when triglycerides were <400 mg/dl.^8^ Glucose was evaluated by the enzymatic method.

### Endothelial function and carotid intima-media thickness

Ultrasound tests were performed to evaluate the subclinical atherosclerosis by carotid intimal medial-thickness (cIMT)^9^ and vasoreactivity assessment of endothelial-dependent flow-mediated dilation (FMD) of the brachial artery.^10^

Briefly, patients were required to fast and abstain from nitrates, alcohol, and vasoactive medications for 24 h prior to the tests. After a 15-20 min rest, the brachial artery in the right antecubital fossa was viewed, using a linear transducer with a frequency of up to 11 MHz, together with simultaneous monitoring by means of an electrocardiogram (ECG). Images were obtained by HP SONOS 5500 ultrasound system (Hewlett Packard, Palo Alto, USA). Once the optimal image of the artery was achieved, the baseline vessel diameter was measured. Reactive hyperemia was induced by inflating the blood pressure cuff to 200 mmHg, or at least 50 mmHg above SBP, on the distal forearm for 5 min and then deflating the cuff. End-diastolic images were obtained at the time of onset of the QRS complex on ECG. These images were acquired at baseline and one min after cuff deflation. The percentage change from the baseline diameter to the value detected during reactive hyperemia was calculated to determine the FMD. The FMD and cIMT measurements were evaluated by an experienced sonographer in a blinded fashion. The intra-sonographer and inter-sonographer variability were less than 1% and 2%, respectively.

### Cytokines and CD4 T-cells

Cytokine concentrations were tested using commercially available ELISA kits. Plasma viral load and CD4 T-cell counts were determined for HIV-infected subjects. The nadir CD4+ T-cell count was defined as the lowest registered and laboratory-confirmed value.

### Autoantigen isolation and synthesis

The LDL particle was obtained from total plasma after centrifugation (1,000 g; 4 °C; 15 min) and supplemented with benzamidine (2 mM), gentamicin (0.5%), chloramphenicol (0.25%), phenylmethylsulfonyl fluoride (PMSF) (0.5 mM), and aprotinin (0.1 unit/mL). Low-density lipoprotein particles (1.006<d<1.063 mg/mL) were isolated by sequential ultracentrifugation (100,000 g; 4 °C), using a rotor (70 Ti, fixe angle; Beckman Coulter, USA) and an ultracentrifuge (Hitachi, Japan). The LDL particle was copper-oxidized and used as an autoantigen to evaluate autoantibodies titers.^11^ The apolipoprotein B-peptides (apoB-peptides) used in this study consisted of two synthetic fragments: ApoB-D (ApoB-D, which is an ApoB-peptide fragment with a 22-amino-acid sequence derivate of domain 3 of the apolipoprotein B sequence in the third conserved portion for tripsin digestions)^12^ and peptide-0033G-Cys (peptide-0033G-Cys, which is a peptide fragment with a 21-amino-acid derivate of domain 3 of the apolipoprotein B sequence in the first conserved portion for trypsin digestion).^12^

### Determinations of autoantibodies

The quantification of oxLDL and apoB-peptide derivate autoantibodies (Abs) was assessed in total plasma by ELISA, as previously described.^13^^,^^14^ Ninety-six-well microtiter plates (Microplates 8096, Costar-USA) were coated with 10µg/mL of the ApoB-D or 0033G-Cys peptide in carbonate/bicarbonate buffer (0.1 mol/l; pH 9.6), which was left for sensitization overnight at 4ºC. After three cycles of washing with phosphate buffered saline (PBS, pH 7.4) plus Tween-20 (0.05%), the plate was blocked with gelatin (3%; room temperature; 24 h). Patients’ plasma samples (50 μl/well, 1:400 in phosphate buffer, PBS, pH 7.4) were added to the plates for 2 hours at room temperature. Next, three more cycles of washing were performed, and secondary IgG horseradish peroxidase-conjugated antibodies (purified goat anti-human IgG, 0.1 µg/ml, KPL, Kirkegaard & Perry Laboratories, Gaithersburg, Maryland, USA) or IgM (purified goat anti-human IgM, 10 µg/ml KPL, Kirkegaard & Perry Laboratories, Gaithersburg, Maryland, USA) were added to evaluate the titers of anti-ApoB-D or anti-0033G-Cys peptide Abs. After incubation (1 hour), the plaques were washed (three cycles), and 3,3,5,5-tetramethylbenzidine (6.5% in dimethyl sulfoxide; Sigma, St Louis, MO) and H_2_O_2_ (Sigma) diluted in citrate/phosphate buffer (0.1mol/l; 250μl; pH 5.5) were added (room temperature), as enzyme substrates. The reaction was stopped by adding H_2_SO_4_ (2mol/l). The optical density (OD) of samples was measured at 450 nm. Autoantibodies (Abs) titers were expressed as the reactivity index (RI), calculated as $\text{RI}=({\text{OD}}_{\text{sample}}–{\text{OD}}_{\text{sample blank}})/({\text{OD}}_{\text{IgG or IgM}}–{\text{OD}}_{\text{IgG or IgM blank}})$ where the IgG or IgM antibodies were used as controls. The intra-assay coefficient of variation was 5.4% and the intra-assay was 2.0%.

Anti-oxLDL Abs titers were performed similarly to the apolipoprotein B peptide assay but using ninety-six-well microtiter plates coated with 7.5 μg/ml of oxLDL.^13^ The total antibodies were determined in total plasma by the ELISA method.

Samples were run in triplicate and the variation within the triplicates did not exceed 5% of the mean.

### Ethics

The study was approved by the institutional ethics committee of the Federal University of São Paulo and University of São Paulo (FO. 99/2009), and written informed consent was obtained from all participants prior to beginning the protocol.

### Statistical analysis

Statistical analyses were performed using the SPSS 17.0 software package (Statistical Package for Social Science, SPSS Inc., Chicago, IL, USA). Categorical variables were compared by Pearson's chi-square test. Distribution of normality was assessed by the Kolmogorov-Smirnov test. Between group analyses were tested by a *t* test or Mann-Whitney test. Interaction between endothelial function and other variables were tested by Pearson or Spearman tests. Variables identified to have significant interaction were tested with stepwise multiple linear regression analyses, with an endothelial function as a dependent variable. A significance level of 5% was used for all tests.

## Results

Clinical and demographic parameters are presented in Table 1. There were no differences in the cIMT among HIV-infected and non-infected subjects. The endothelial function was decreased in HIV-infected subjects (p=0.040). (Table 1).

**Table 1:** Characteristics of the HIV naïve infected and uninfected subjects

Variables		Overall (93)	HIV- (Control) (53)	HIV+ (Naïve) (40)	p-values
**Clinical parameters**					
	Gender (males/females)	63/30	32/21	31/9	0.110
	Age (years)	32 (1.0)	32 (1.7)	32 (1.3)	0.746
	Abdominal circumference (cm)	88 (83-97)	89.5 (76.5-100)	97 (83-96)	0.668
	Body mass index (kg/m²)	24.8 (23-28)	25.5 (21.5-28.5)	25.2 (23.5-28)	0.586
	Smokers (%)	11	6	5	0.951
	Systolic blood pressure (mmHg)	120 (110-120)	120 (110-120)	120 (110-120)	0.631
	Diastolic blood pressure (mmHg)	80 (70-80)	80 (70-80)	80 (70-80)	0.441
**Biochemical analysis**					
	Total cholesterol (mg/dL)	165 (139-185)	166 (144-191)	150 (124-176)	0.028
	LDL-c (mg/dL)	98 (69-115)	103 (77-119)	91 (66-113)	0.095
	HDL-c (mg/dL)	46 (37-65)	47 (40-57)	36 (30-46)	0.008
	Triglycerides (mg/dL)	91 (52-122)	88 (67-131)	113 (73-131)	0.285
	Glucose (mg/dL)	90 (86-94)	90 (86-94)	92 (86-96)	0.425
**HIV parameters of infection**					
	Time of infection (years)	−	−	3 (1-6)	N.A
	CD4 count (cells/µL)	−	−	447 (366-590)	N.A
	CD4 nadir (cells/µL)	−	−	402 (356-537)	N.A
	HIV viral load (RNA copies/µL)	−	−	2623 (485-26225)	N.A
	HBV coinfection	0	0	4	N.A
	HCV coinfection	0	0	3	N.A
**Therapies in use**					
	Antihypertensive (individuals, *N*)	4	3	1	N.A
	Statins (individuals, *N*)	0	0	0	N.A
	Neurological drugs (individuals, *N*)	3	2	1	N.A
**Inflammatory markers**					
	hs-CRP (mg/L)	1.20 (0.30-1.92)	0.51 (0.20-1.87)	1.48 (0.82-3.30)	0.017
	IFN-γ (pg/dL)	2.84 (0.90-6.85)	1.43 (0.87-4.10)	3.89 (1.30-8.85)	0.021
	TNF-α (pg/dL)	6.66 (5.58-7.31)	6.02 (5.51-6.94)	6.90 (6.54-7.63)	0.020
	IL-6 (pg/dL)	1.54 (1.37-1.80)	1.54 (1.36-1.63)	1.50 (1.37-1.95)	0.028
	IL-8 (pg/dL)	3.13 (2.50-4.60)	2.80 (2.20-4.40)	3.65 (2.70-5.50)	0.050
	IL-10 (pg/dL)	1.75 (0.39-1.97)	1.79 (0.80-1.98)	0.87 (0.36-1.94)	0.088
**Total antibodies**					
IgG total serum (RI)		1.33 (1.19-1.38)	1.34 (1.20-1.38)	1.33 (1.18-1.37)	0.877
IgM total serum (RI)		0.69 (0.55-0.84)	0.67 (0.49-0.82)	0.73(0.58-0.86)	0.310
**Subclinical atherosclerosis**					
	Intima-media Thickness (mm)	0.67 (0.57-0.68)	0.67 (0.56-0.68)	0.67 (0.57-0.68)	0.971
**Endothelial function**					
	Flow-mediated Dilatation (%)	11.6 (1.4)	13.7 (2.4)	9.3 (1.2)	0.040

HBV: hepatitis B virus; HCV: hepatitis C virus; N.A: not applicable; RI: reactivity index.

As expected, HIV-infected subjects had significantly higher inflammatory marker levels than did non-infected individuals; however, the anti-inflammatory cytokine IL-10 did not differ between the groups (Table 1).

Serum total IgG and IgM Abs titers did not differ among HIV-infected and uninfected subjects (Table 1). Figure 1 demonstrated that titers of IgG anti-oxLDL Abs were higher in HIV-infected subjects (p<0.001). However, the titers of IgM anti-oxLDL Abs did not differ among HIV-infected and uninfected subjects. HIV-infected subjects had higher titers of IgG anti-ApoB-D Abs (p<0.001) and lower titers of IgM anti-ApoB-D when compared to non-infected subjects (p=0.040). No differences were observed among groups for the anti-peptide-0033G-Cys Abs.

**Figure 1:** Humoral response to oxidized LDL, ApoB-D peptide and 0033-peptide in HIV-infected patients and non-infected controls. (A) IgG anti-oxLDL autoantibodies (Abs); (B) IgM anti-oxLDL Abs; (C) IgG anti-ApoB-D peptide Abs; (D) IgM anti-ApoB-D Abs. (E) IgG anti-0033-peptide Abs; (F) IgM anti-0033-peptide. Significant differences between groups were calculated by the Mann-Whitney test.

The present study showed that in HIV-infected subjects the endothelial function was associated with IgM anti-ApoB-D Abs titers [β=10.75; p=0.015] (Table 2). The stepwise regression model, including traditional cardiovascular risk factors, HIV-related markers, and immune responses showed that IgM anti-ApoB-D Abs were associated with the endothelial function [β=7.28; p=0.002]. Associations among IgG anti-ApoB-D and the endothelial function were not observed. Regarding subclinical atherosclerosis, the cIMT measures were not associated with the humoral response for both peptides.

**Table 2:** Univariate adjusted analysis of potential risk factors associated with endothelial function in HIV-infected subjects

Variables		
	β	p-values
Age (years)	-0.187	0.350
Abdominal circumference	-0.049	0.708
IgM anti-ApoB-D peptide	10.754	0.015
IgG anti-ApoB-D peptide	0.597	0.351
Nadir CD4	0.007	0.135
Current CD4	-0.010	0.126
Log viral load	0.413	0.786
Time of infection	-0.215	0.718

β-coefficient represent the changes in the percentage of flow-mediated dilatation for the predictor variables. Adjustments were made for hypertension, current smoking, and dyslipidemia. CI: Confidence interval.

## Discussion

The present study showed that, in HIV-infected subjects, naïve of antiretroviral therapy, a reduced endothelial function accompanies distinct modulation in Abs against apoB-peptides fragments, as compared to non-infected subjects, regardless of serum total Abs titers.

Data related to humoral immunity and apoB peptides suggest that their presence is associated with atherosclerotic disease progression, as part of an autoimmune response.^5^^,^^14^ However, these Abs can participate in the clearance of pro-atherogenic products generated from the oxidation of LDL particles and the modification of apoB, performing a dual role in the atherogenesis process.^15^^,^^16^

This study also showed that IgM Abs against ApoB-D were associated with ED, corroborating with previous studies.^6^^,^^17^ Our findings suggest that there is a clearance of apoB autoantigens by natural antibodies, suggesting that they may be involved in vascular repair after an injury process,^18^ however the effects of HIV infection on FMD may be attributable to a distinct stage of disease and distinct drug therapies adopted.^19^

Cohort studies and meta-analysis showed that cIMT in HIV-infected, when compared to non-infected, subjects is higher.^20^ We believe the time of infection in our study was not enough to promote carotid atherosclerosis modification detected by an ultrasound exam.

The present study's results suggest that autoantibodies to defined-apoB peptides can be a marker of endothelial dysfunction, or even of an elevated inflammatory response, but not of carotid atherosclerosis in HIV-infected patients. Cohort and clinical trials with patients submitted to HARRT merit further investigation to confirm these preliminary results.

The cross-sectional design and the lack of a group receiving antiretroviral therapy for comparisons of the effects of HAART drugs on endothelial function and subclinical atherosclerosis are a limitation. No significant differences were found among sex groups, which may be justified by the small number of subjects included in this study. Additional studies, including a larger number of patients, are needed to confirm our findings related to sex, infection, and endothelial function. For adjustments, the effects of different cardiovascular risk factors and infection markers were evaluated as a possible explanation for the observed natural immune response associated with the vascular function.

## Conclusion

This study's findings suggest that natural immunity to apoB antigens is associated with ED. Further prospective studies for the evaluation of HIV immunological parameters in autoimmune response and these effects on vascular function are warranted.
